# Informing new or improved vector control tools for reducing the malaria burden in Tanzania: a qualitative exploration of perceptions of mosquitoes and methods for their control among the residents of Dar es Salaam

**DOI:** 10.1186/s12936-017-2056-9

**Published:** 2017-10-11

**Authors:** Christina Makungu, Stephania Stephen, Salome Kumburu, Nicodem J. Govella, Stefan Dongus, Zoe Jane-Lara Hildon, Gerry F. Killeen, Caroline Jones

**Affiliations:** 1 Environmental Health and Ecological Sciences Department, Ifakara Health Institute, Kiko Avenue, Mikocheni, PO Box 78373, Dar Es Salaam, United Republic of Tanzania; 2 Vector Biology Department, Liverpool School of Tropical Medicine, Pembroke Place, Liverpool, L35QA UK; 3 Department of Epidemiology and Public Health, Swiss Tropical and Public Health Institute, Socinstrasse 57, P.O. Box 4002, Basel, Switzerland; 4 University of Basel, Petersplatz 1, 4003 Basel, Switzerland; 5 KEMRI-Wellcome Trust Research Programme, Kilifi, Kenya; 6 London School of Hygiene and Tropical Medicine, London, UK; 7 Johns Hopkins Center for Communication Programs, Johns Hopkins University, Baltimore, USA

**Keywords:** Mosquito, Malaria, Community perceptions, Qualitative, Photovoice, Bed net, Repellent, Larval source management

## Abstract

**Background:**

The effectiveness of malaria prevention with long-lasting insecticidal nets and indoor residual spraying is limited by emerging insecticide resistance, evasive mosquito behaviours that include outdoor biting, sub-optimal implementation and inappropriate use. New vector control interventions are required and their potential effectiveness will be enhanced if existing household perceptions and practices are integrated into intervention design.

**Methods:**

This qualitative descriptive study used focus groups discussions, in-depth interviews and photovoice methods to explore mosquito control perceptions and practices among residents in four study sites in Dar es Salaam, Tanzania.

**Results:**

Mosquitoes were perceived as a growing problem, directly attributed to widespread environmental deterioration and lack of effective mosquito control interventions. Malaria and nuisance biting were perceived as the main problem caused by mosquitoes. Breeding sites were clearly distinguished from resting sites but residents did not differentiate between habitats producing malaria vector mosquitoes and others producing mostly nuisance mosquitoes. The most frequently mentioned protection methods in the wealthiest locations were bed nets, aerosol insecticide sprays, window screens, and fumigation, while bed nets were most frequently mentioned and described as ‘part of the culture’ in the least wealthy locations. Mosquito-proofed housing was consistently viewed as desirable, but considered unaffordable outside wealthiest locations. Slapping and covering up with clothing were most commonly used to prevent biting outdoors. Despite their utility outdoors, topical repellents applied to the skin were considered expensive, and viewed with suspicion due to perceived side effects. Improving the local environment was the preferred method for preventing outdoor biting. Affordability, effectiveness, availability, practicality, as well as social influences, such as government recommendations, socialization and internalization (familiarization and habit) were described as key factors influencing uptake.

**Conclusions:**

Outdoor transmission is widely accepted as an obstacle to malaria elimination. Larval source management, targeting both malaria vectors and nuisance-biting mosquitoes, is the preferred method for mosquito control among the residents of Dar es Salaam and should be prioritized for development alongside new methods for outdoor personal protection. Even if made available, effective and affordable, these additional interventions may require time and user experience to achieve positive reputations and trustworthiness.

**Electronic supplementary material:**

The online version of this article (doi:10.1186/s12936-017-2056-9) contains supplementary material, which is available to authorized users.

## Background

The scale-up of effective malaria prevention and treatment tools, such as long-lasting insecticidal nets (LLINs), indoor residual spraying (IRS), rapid diagnostic tests (RDTs) and artemisinin-based combination therapy (ACT) have substantially reduced the malaria burden across malaria-endemic countries, especially in Africa [1]. Nevertheless, it has been estimated that in 2015 there were still 214 million cases of malaria globally and 438,000 malaria deaths, of which 89% of cases and 91% of deaths occurred in sub-Saharan Africa (SSA) [2]. While malaria remains a major public health challenge in SSA the physiological resistance of mosquitoes to insecticides is undermining the effectiveness of the core vector control interventions, specifically LLINs and IRS [3]. Furthermore, the impact of LLINs and IRS is fundamentally limited by mosquito behaviour that allows them to evade contact with their insecticidal active ingredients, notably feeding and resting outdoors [4]. There is increasing evidence that malaria transmission can persist despite the widespread use of LLINs, IRS and mosquito proofed housing [4–8].

As with any public health intervention, the efficacy of LLINs, IRS and mosquito-proofed housing depends not only on the behaviour of the mosquitoes, but also the behaviour of humans [6]. Even efficacious interventions such as LLINs and IRS are unlikely to be effective for all groups in all communities at all times. For example, many people undertake activities that prevent them from being under a LLIN at the times they are at risk from malaria (e.g., getting up before dawn to get to market or collect wood), or sleep in locations where they are not protected by LLINs due to socio-economic circumstances, climatic obstacles, cultural practices, or personal preferences (e.g., visiting relatives or seasonal migration to farm) [9, 10]. The most obvious of the behaviour known to mediate such *residual* malaria transmission is outdoor biting in the early evening and/or early morning; behaviour that clearly limits the effectiveness of interventions focused on the prevention of indoor biting [11–13]. These long-standing challenges will clearly require complementary additional vector control tools in order to eliminate transmission in many settings [11–13]. However, maximizing the potential effectiveness of any intervention (optimal implementation, uptake and use) requires that the contexts within which it will be implemented, in particular the existing perceptions and practices of target communities, are integrated into the intervention design process [14, 15].

This paper reports the results of a study undertaken in Tanzania to explore the factors influencing the uptake and use of vector control interventions by householders across a range of socio-economic contexts in and around the city of Dar es Salaam. The specific questions the study sought to answer were:

1. What are the current perceptions of mosquitoes among householders in Dar es Salaam?
2. What protection measures do householders currently employ against mosquitoes?
3. What factors influence the uptake of protection measures against mosquito bites?

## Methods

The study was based on a social constructivist approach, focusing on understanding the participants’ views and the meaning they ascribe to their experiences [16]. The design was exploratory using three complementary qualitative and participatory methods, to enable data triangulation across independent methods: photovoice (PV), focus group discussions (FGDs), and in-depth interviews (IDIs). PV is a photographic approach to documenting user perceptions that is emerging as a new tool in malaria research [17, 18]. It is a participatory research method which allows participants to identify, represent and document objects, processes and phenomena within their community through photography [19, 20]. The method enables participants to record and reflect their community’s strength and concerns, to promote critical dialogue and knowledge through group discussions, and to communicate with policy makers [19, 20]. The PV approach involves a series of procedural steps that guide the ethical implementation of the method [17–20]. The results are reported according to the criteria for reporting qualitative research [21].

## Study setting

The study was carried out in and around Dar es Salaam, the largest city and commercial centre of the United Republic of Tanzania, located along the shores of the Indian Ocean with a hot and humid climate [22]. Dar es Salaam is a typical coastal African city, with ideal climatic conditions for malaria transmission, where *Plasmodium falciparum* is transmitted both indoors and outdoors [22, 23]. There are typically two rainy seasons: a main rainy season from March to June and a shorter, more erratic rainy season from October to December [22]. The Dar es Salaam region has 4.4 million inhabitants [24] with an average annual growth rate of 5.6% [25] making it the third fastest-growing city in Africa and the tenth fastest in the world [26]. This rapid and unprecedented urbanization is associated with unplanned settlements, resulting in about 70% of the inhabitants living in informal settlements [26]. Poor drainage and sewage systems, as well as overloaded solid waste collection systems, lead to regular flooding in many parts of the city [24, 27]. All these factors exacerbate malaria transmission, by providing ideal conditions for mosquitoes to breed in stagnant surface water, and also exacerbate vulnerability to transmission exposure amongst residents by creating difficult living conditions that limit household resilience [27]. The municipal local government, with support and supervision from the National Malaria Control Programme, currently implements all organized malaria vector control interventions in Dar es Salaam. At the time of the study, these interventions included free LLINs to all sleeping spaces and weekly larvicide application to *Anopheles* habitats and environmental management [5, 28–30].

Additionally, Dar es Salaam has experienced remarkably rapid, spontaneous scale-up of mosquito-proofed housing over recent years, entirely implemented and self-funded by residents of the city, with protection against mosquitoes as their most important motivation [5, 31]. These activities have resulted in substantive reduction of malaria prevalence [5, 22, 28, 31–33] but local malaria transmission persists, with malaria infection risk known to be influenced by human behaviour that exposes individuals to outdoor transmission in the evenings and mornings [5].

Administratively, Dar es Salaam city has three municipalities: Ilala, Kinondoni and Temeke, which in turn sub-divide into 90 wards spanning the full range of urban, rural and mixed environments, at the time [34, 35]. In the Tanzanian governmental administration system, wards are further divided into smaller neighbourhood units called *mitaa* (a Kiswahili word for street, written in a singular form as *mtaa*) in urban areas or *vijiji* (villages) in rural areas [35]. *Mitaa* are sub-divided into 10 cell units or clusters (TCUs), which are the smallest units of local government, headed by a locally elected representative known as a *balozi* or *mjumbe* [22]. TCUs are typically comprised of approximately 10 to 20 houses each, but some TCUs contain much larger numbers of houses [36].

**Fig. 1:**
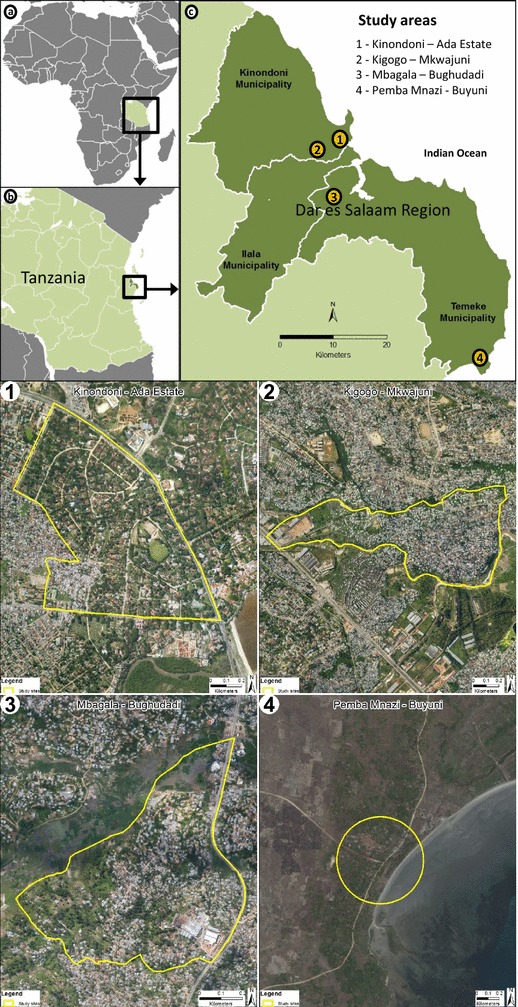
Map of the study area. **a** Map of Africa showing location of Tanzania (box), **b** map of Tanzania showing location of Dar es Salaam Region (box), **c** map of Dar es Salaam Region showing location of the four study sites. 1 Kinondoni-Ada Estate (urban study site), 2 Kigogo-Mkwajuni (urban study site), 3 Mbagala-Bughudadi (peri-urban study site), 4 Pemba Mnazi-Buyuni (rural study site)

This study was conducted at four distinct locations in *mitaa* distributed widely across the Dar es Salaam region: Ada Estate in Kinondoni ward, Mkwajuni in Kigogo ward, Bughudadi in Mbagala ward, and Buyuni in Pemba Mnazi ward (Fig. 1). These areas represent different levels of urbanization: Kinondoni Ada Estate and Kigogo Mkwajuni are both urban, while Mbagala Bughudadi is peri-urban and Pemba Mnazi Buyuni is essentially rural (Fig. 1). Geography, land use type, population density and socio-economic status, as well the research team’s experience [5, 34, 37–39] of the city were all considered in the selection of these study locations. Ada Estate is a relatively high-income, urban location with a planned, low-density settlement pattern (Fig. 1, location 1), where low densities of *Anopheles* and moderate densities of *Culex* mosquitoes occur because of proximity to Msimbazi River. Kigogo Mkwajuni (urban) and Mbagala Bughudadi (peri-urban) are both densely populated informal, unplanned settlements (Fig. 1, locations 2 and 3, respectively), bordering rivers that regularly flood during the rainy season. Mbagala Bughudadi is close to a lagoon near the Kizinga River valley, with lots of agriculture activities and moderate to high mosquito densities. Kigogo Mkwajuni is located very centrally at the edge of the Msimbazi River valley, the largest flood plain in the city, and has high mosquito densities. Pemba Mnazi, although administratively part of the Dar es Salaam city region, is very rural in character, with only a few small, scattered houses, some of them with thatched roofs (Fig. 1, location 4). It is approximately 70 km southeast of Dar es Salaam, where fishing and some agriculture are the main income-generating activities. It is close to coastal lagoon and mangrove habitats, as well as some natural drainage lines.

## Study participants

The primary inclusion criterion for study participation was being an adult (18 years or older) household member who lived within one of the study locations and who consented to participate after having been informed of the purpose and procedures of the study, as well as their right to refuse or withdraw at any time. Participants were purposively sampled to ensure representation by age (classified as either younger adults of 18–25 years or older adults of 26–60 years) and gender. For the selection of PV participants, familiarity, integrity and trustworthiness of participants in the eyes of community members was an important additional criterion as these participants were involved in taking photographs in both public and private places. All study participants were, therefore, identified and recruited with the help of *mtaa*-level local government leaders. In this study, a total of 32 PV participants (photographers) were recruited, 2 men and 2 women in each study location in phase one (total n = 16), and 4 participants in each study location in phase two (total n = 16). For the community FGDs, 8–12 people participated in each FGD.

## Data collection methods

To explore if perceptions and practices relating to mosquitoes varied with changing seasons, all data collection activities were conducted in two phases: during the rainy season between March and May 2012 and repeated during the dry season between August and September 2012 (Table 1).

**Table 1:** Study locations and data collection methods

Location	Characteristics	Season	FGD number held	IDI number held	PVGD number held
Kigogo Mkwajuni	Urban, low income	Rainy	4	8	1
		Dry	3	8	1
Ada Estate	Urban, high income	Rainy	2	8	1
		Dry	0	8	1
Mbagala Bughudadi	Peri-urban, middle income	Rainy	4	8	1
		Dry	3	8	1
Pemba Mnazi	Rural, low income	Rainy	4	8	1
		Dry	3	6	1
Total			23	62	8

*FGD* focus group discussion, *IDI* in-depth interview, *PVGD* photovoice group discussion

## Photovoice

After recruitment, the PV participants were introduced to the concept and methods to be used. They were then familiarized with underlying issues relating to the basics of camera use, as well as the ethics of photographic reporting, notably potential risks and how to minimize these risks. The PV participants (photographers) signed written informed consent forms, which included ethical conduct of photo-taking, a statement of project activities and significance before they undertook any PV activities (Additional file 3). The photographers were then provided with disposable cameras and given 2 weeks to take photographs of things they associated with mosquitoes. No specific thematic orientation was given to them, and they were asked to take pictures within the community while respecting the privacy of other community members. After 2 weeks, the photographers returned the cameras to the research team who arranged for the pictures to be developed. Once the pictures were developed, photographers were engaged in a two-stage process of participatory analysis; selecting photographs for discussion and then contextualizing or storytelling. In the first stage, developed pictures were given back to photographers, each of whom was given approximately 1 week to select what he/she considered to be his/her 10 best or most significant photographs. By selecting photographs for discussion, participants led the overall direction of subsequent PV group discussions (PVGDs) [40]. The second stage consisted of contextualizing or telling stories about what the photograph meant to the photographer, during the PVGD. PVGDs were then organized in each location with the local group of photographers. Each participant displayed his/her photographs on the table, introduced them to the group, narrated the meaning of his/her photographs, and explained how the images were associated with mosquitoes (Additional file 1). These PVGDs were conducted informally, but based on an adapted version of the SHOWeD model [20]. At this stage of the discussion, each photographer identified different themes that emerged after re-examining the contents of their photographs and remembering where, when and why they took them. This was followed by a more specific discussion (guided by a topic guide) of perceptions of mosquitoes, methods of protection against mosquitoes, and factors influencing their use (Additional file 2). At the end of the discussion the PVGD participants selected the 10 best pictures out of all of the photographs taken in their area, for use in subsequent community FGDs and householder in-depth interviews. All interviews and group discussions with the photographers were conducted in *kiSwahili* (the local language) and with the permission of the participants, digital audio recordings were made. These recordings were subsequently transcribed verbatim (with identifiers removed), and translated into English, as Microsoft Word^®^ documents.

## FGDs and IDIs

Subsequent to the PV activities, FGDs with community members were held in *mtaa* local government offices, or in the home compound of a participant. The FGDs were conducted in *kiSwahili* (Additional file 2), with each discussion lasting for between one-and-a-half and two hours. With permission of the participants, the discussions were audio digitally recorded. In three of the study locations (Kigogo Mkwajuni, Mbagala Bughudadi, Pemba Mnazi), four FGDs were conducted per location during the rainy season (one each with younger women, older women, younger men and older men) and three FGDs (one with older women and one older men and one group combined both younger men and women) per location during the dry season (exactly which three categories varied by location). In the Ada Estate area, a quite affluent area, it proved very difficult to recruit people to take part in an FGD, so only two FGDs were conducted during the rainy season, with each group combining men or women of both age groups (older and younger, together). No FGDs were conducted in this area during the dry season. Potential participants in this location preferred to be interviewed in their own home and at a time of their own convenience, rather than gathering with other participants in *mtaa* government offices or other participants’ compounds. The data for the high-income location are therefore based primarily on individual IDIs and the PVGDs in that area.

During both IDIs and FGDs, participants were shown the PV pictures, which were displayed on the table, or pasted on the wall, asked if they associated any of them with mosquitoes, and then asked to explain why. During the subsequent discussions/interviews, the participants were asked about their perceptions of mosquitoes, including where mosquitoes come from and the population groups they considered to be most vulnerable to the problems caused by mosquitoes. In addition, questions were asked about perceptions of current measures available for protecting against mosquito bites in indoor and outdoor environments, as well as factors influencing their uptake (Additional file 2). All FGDs and IDIs were audio recorded, transcribed verbatim and translated into English.

## Research team and reflexivity

Prior to data collection, two experienced research assistants who are fluent in *kiSwahili* (SK and SS) were recruited and trained on appropriate approaches to probing, data confidentiality and data management. The first author (CM) was the team leader who has experience in conducting qualitative research. She conducted most of FGDs and PVGDs. SK and SS assisted in conducted fieldwork and contributed in preliminary analysis of data, with their roles including recruitment of study participants, seeking informed consent, and writing field notes. Study participants did not know the interviewers, who were introduced on the day of the data collection by *Mtaa* leaders.

## Data processing and analyses

The data from the PV discussions, FGDs and IDIs were analysed using a framework approach, in which both pre-determined codes following the main topic areas included in the discussion guides (inductive coding), and emergent codes to capture new themes that arose during analysis (deductive coding) were applied [41]. After initial coding of all transcripts, the next step was to look for similarities and differences between patterns and themes. Relationships and connections between themes were established and the final step was the interpretation of data.

## Ethics, consent and permissions

No identifiable personal data were requested during the PV, FGDs or IDIs, and any shared inadvertently was excluded from the anonymized subset of data reported herein. All photographs presented in Fig. 5 which included the faces of individuals were anonymized by screening their identifiable facial features. Ethical approval was secured from the Ifakara Health Institute Institutional Review Board (IHI/IRB/NO:26-2011) and National Institute of Medical Research (NIMR/HQ/R.8a/Vol.IX/1236). All participants were informed of the objectives, procedures, risks and benefits of the study, as well as their right to decline or withdraw from participation. Informed consent was documented in writing (see Additional files 1, 2, 3, 4, 5).

## Results

The presentation of the results is structured to reflect the three major themes that were defined a priori by the research questions: (1) what are the current perceptions of mosquitoes among householders in Dar es Salaam?; (2) what protection measures do householders currently employ against mosquitoes?; and, (3) what factors influence the uptake of protection measures against mosquito bites?

## Perceptions of mosquitoes

Despite the significant differences in socio-economic status and environmental surroundings between the four-study locations, there was no obvious variation in the perceptions of mosquitoes regarding types of mosquitoes, problems caused by mosquitoes, or the locations of potential breeding/resting sites.

## Mosquito types, biting nuisance and mosquito-borne diseases

For most participants, a mosquito was a mosquito, and few were able to distinguish between different types of mosquitoes or the different diseases they transmit. The names *Anopheles* and *Culex* were sometimes mentioned, but no participant commented on which kind was more common. Among those participants who did mention that were differences, distinction among adult mosquitoes was made by their colour, shape, noise they make, and the places where they were found.

*‘Some mosquitoes have spots, they have various colours, they are small, they cause much itching when they bite. They are known as ‘suni’.’* (Male, FGD participant, peri-urban, low income).

Nuisance biting and malaria were unambiguously cited by the majority of participants as the main problems caused by mosquitoes, whilst elephantiasis and yellow fever were also mentioned by some participants. Across all locations, malaria was perceived to be closely associated with mosquitoes. Malaria was viewed by the majority of participants as the most threatening disease caused by mosquitoes, because of its recurrence, severity and the costs of prevention and treatment.

*‘When I feel sick, I must go for a check*-*up. When they find malaria, I take the treatment until I finish. I may feel okay for some time, but after 2 or 3* *weeks. I start to feel sick again. They would say you have two parasites again after diagnosis. When you get relief from malaria, it doesn’t take long before you fall sick again.’* (Female, FGD participant; rural, low income)

The majority of participants viewed mosquitoes as a growing problem in Dar es Salaam, and associated increased mosquito populations with wider environmental deterioration caused by urbanization and lack of effective mosquito control interventions. Overcrowding, lack of adequate urban planning, drainage and ineffective waste disposal management, combined with lack of sufficient understanding of mosquito exposure risk behaviours among city dwellers, were also perceived by participants to be associated with increased densities of mosquitoes.

*‘Nowadays environmental pollution is increasing if you compare with previous years. Mosquitoes have increased a lot because of human activities. Some people are building their houses on top of water drains, water drains are blocked with no water flowing, so mosquitoes breed. High [mosquito] population, combined with human activities and behaviours and ineffective garbage collection, make the situation doubly worse.’* (Male, IDI, peri urban, low income)

## Mosquito breeding sites

**Fig. 2:**
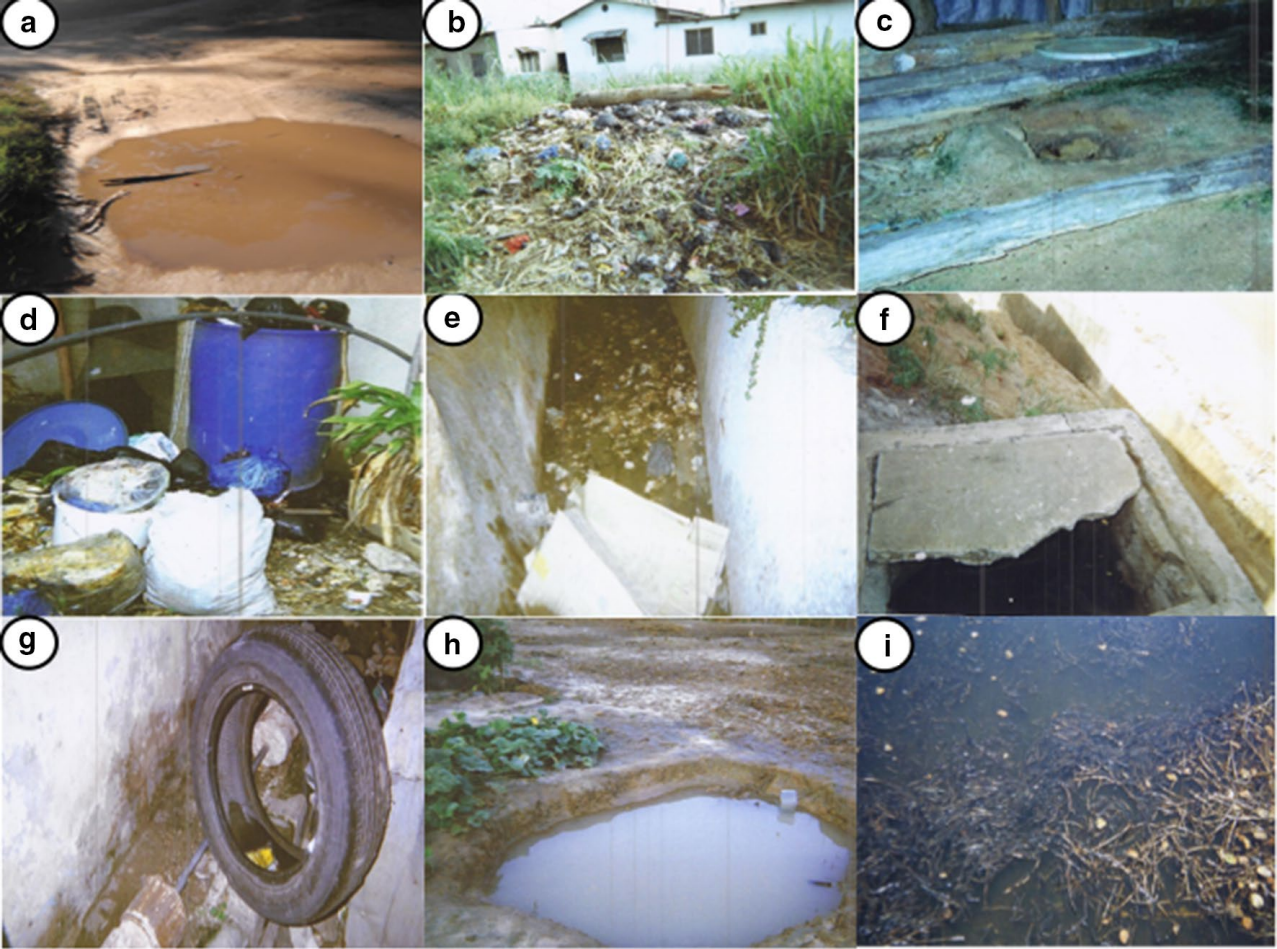
Photographs taken by community participants of perceived mosquito breeding sites. **a** A puddle with dirty stagnant water, **b** rubbish, **c** pit latrine, **d** dustbins containing water, **e** unmaintained drain, **f** uncovered septic tank, **g** discarded tyre, **i** shallow wells used for irrigation, **h** tidal shore near the sea

Pictures taken by PV participants and perspectives shared by FGD and IDI participants consistently indicated that most people differentiated between mosquito breeding sites and mosquito resting sites. Pictures of mosquito breeding sites were primarily of all kinds of stagnant water, particularly dirty stagnant water (Fig. 2), which includes man-made habitats and natural habitats. Across all study locations, most participants considered that human activities contributed significantly to the creation of mosquito breeding sites. Man-made habitats such as puddles, blocked storm water drains, pit latrines, uncovered septic tanks, discarded tyre, discarded tins and coconut shells, brick-making holes, houses under constructions and shallow wells used for irrigation were frequently photographed and mentioned as mosquito breeding sites. The most frequently photographed and mentioned natural habitats were ponds, puddles and tidal marshes near the sea, while some participants also mentioned riverbanks. None of the participants, including those who named different types of mosquitoes, distinguished between the breeding sites of different kinds of mosquitoes.

**Fig. 3:**
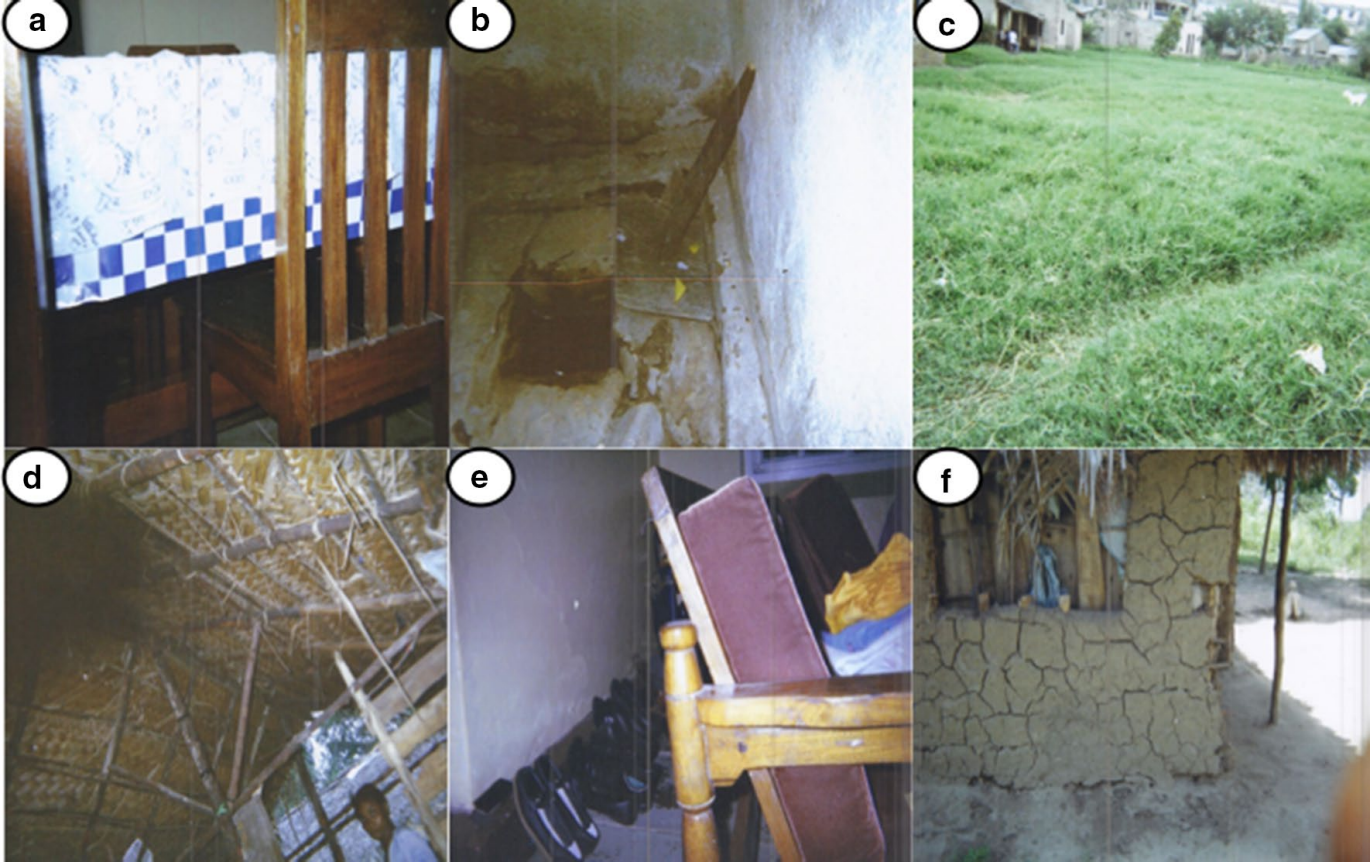
Photographs taken by community participants of perceived mosquito resting sites. **a** under a table (dark area), **b** pit latrine, **c** dense vegetation, **d** thatched roof, **e** shoes, **f** cracked wall

By contrast to the wetness associated with breeding sites, the most common feature associated with resting sites for mosquitoes was darkness. Pit latrines, unattended room, sheltered places without water, such as shoes, thatched roofs, cracked walls and vegetation were described as hiding places for mosquitoes. These dark, sheltered habitats were the major focus of pictures taken that were confirmed to be considered as mosquito resting places in FGDs and the IDIs (Fig. 3). Other non-aquatic habitats, such as less dense vegetation like flowers, bushes and trees, or dirt and rubbish inside or outside of houses, were also frequently mentioned by participants as sources of mosquitoes, that is, places they emerged from after resting.

## Perceptions of available measures for protection against mosquito bites

**Fig. 4:**
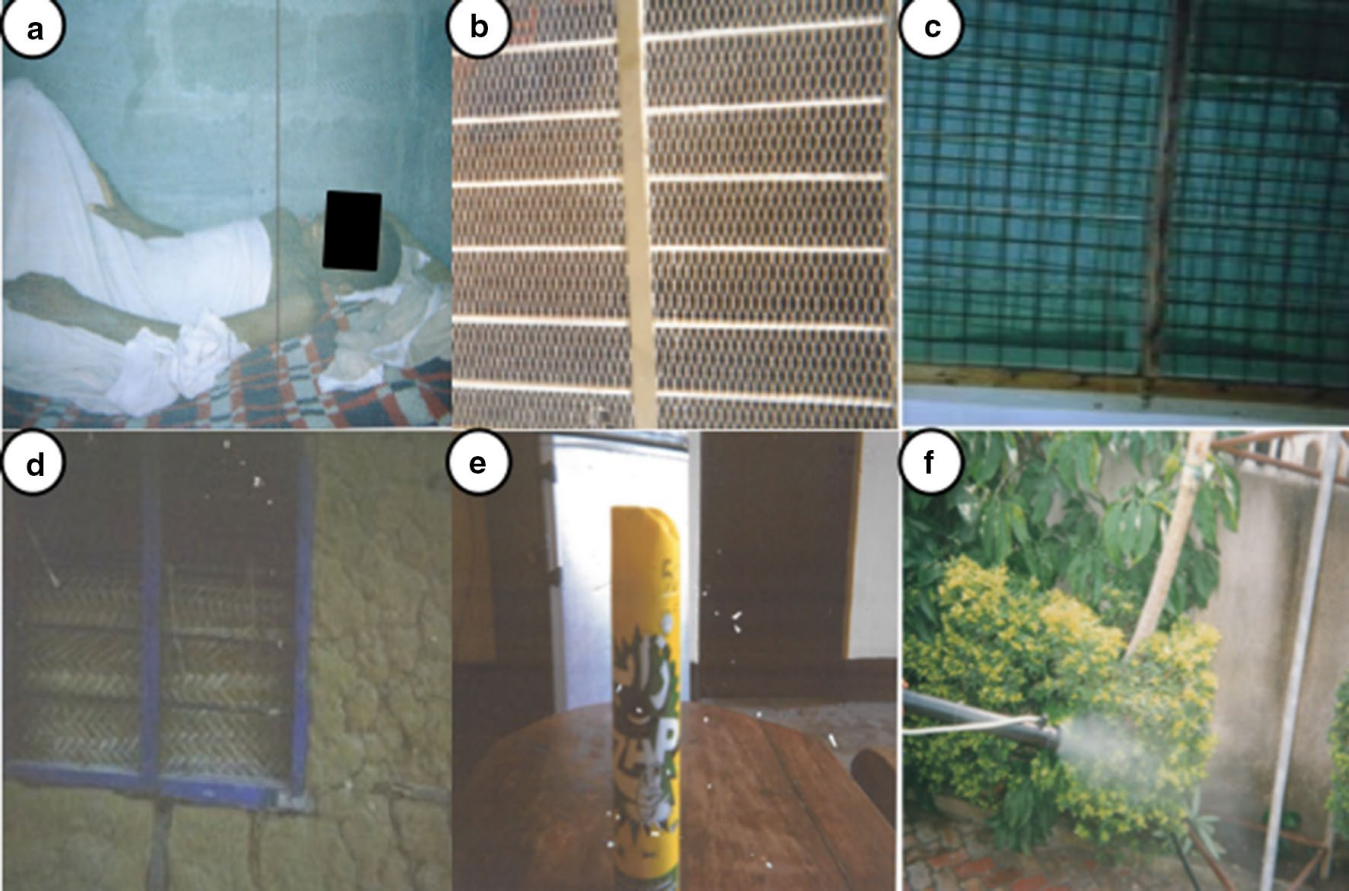
Photographs taken by community participants of perceived mosquito protection measures. **a** Sleeping under a bed net, **b** netting window screens on a house, **c** netting window screens on a house, **d** window screened with thatch, **e** insecticide spray, **f** application of garden pesticides

In contrast to the lack of variation in perceptions of mosquito breeding and resting sites and the nuisance that they cause among the four study sites, there was considerable variation in the use of different methods for protection against biting mosquitoes. Across all study locations, LLINs were by far the most commonly mentioned method of protection against mosquito bites while in bed (Fig. 4). However, there were significant differences in the extent to which residents of different study locations said that in practice they relied on LLINs to protect them against mosquito bites. In the high-income setting, all participants reported using additional methods for protection and some of the participants said that they did not use LLINs because their houses were adequately sealed against mosquito entry. Mosquito-proofed housing and insecticide sprays were commonly mentioned among this group (Fig. 4), while skin repellents and mosquito coils were also mentioned as being more selectively used on specific occasions. The following statement illustrates how residents of the highest income, well-planned settlement protect themselves with multiple interventions indoors, but perceive a lack of options for protecting themselves while outdoors:

*‘I know other people use also bed nets in Ada Estate, but in my house we do not use them because my house is well sealed, with window screens and ceiling boards. We have used these for years! Due to carelessness, sometimes a few mosquitoes may enter inside the house so we normally use sprays. We normally fight with mosquitoes when we are outside the house.’* (Male, IDI respondent, urban, high income)

In peri-urban and urban locations with lower income levels, the majority of participants said that they relied mostly on LLINs to protect themselves from mosquitoes, although some reported using additional methods, such as mosquito-proofed housing and insecticidal sprays. The use of fans, topical skin repellents, mosquito coils, bed sheets, and electric racquets were also mentioned by a small number of participants in all urban and peri-urban locations. Commercial pest control services for domestic residences, to eliminate pests including cockroaches, flies and mosquitoes, were also mentioned by many of the participants in the urban and peri-urban locations as an option for protection. According to participants, such activities are organized by *Mtaa* government offices and implemented by private-sector fumigation companies, with residents paying between 2000 Tanzanian shillings (equivalent to US$0.90) for modern toilets and 1000 shillings (equivalent to US$0.45) for a pit latrine per visit. Almost all study participants from the study locations where these fumigation activities were undertaken expressed dissatisfaction with the service in terms of their impact upon mosquitoes.

*‘I think these people (fumigation companies) use fake chemicals because nothing happens to mosquitoes after fumigation! It does not kill mosquitoes at all.’* (Male, IDI respondent, urban, low income)

In the rural location, the use of private fumigation companies was never mentioned and LLINs were universally described as almost the only form of protection available, with only a few houses having windows with mosquito-proof netting. Participants who relied on only LLINs as a protection measure reported that indoor exposure to biting mosquitoes was still as important a problem as outdoor exposure, specifically exposure which occurs while awake outside of their beds and LLINs, such as in sitting rooms.

*‘We are normally bitten by mosquitoes outside of the bed. We get some relief in bed, but sometimes we spend time watching TV until 11.00* *pm in the sitting room or sometimes we sleep outside on a mat after having their dinner, where we are bitten by mosquitoes because we have nothing to protect ourselves outside.’* (Female, FGD participant, peri urban, low income)

Across all locations, LLINs were described by the majority of participants as being part of “our culture” but their effectiveness as a means of malaria prevention was frequently questioned.

*‘There are many diseases …but the most common disease is malaria. Although we use bed nets, still malaria continues to be a problem in our area.’* (Male, FGD participant, urban, low income)

Specifically, the restriction of their utility to indoor sleeping spaces at night was frequently mentioned as a limitation.

*‘It is only bedtime when we feel comfortable! Outside the bed, it is terrible, and mosquitoes bite a lot. As I have said, during evening time we have no means of controlling them other than bed nets [in beds].’* (Female, IDI respondent, rural, low income)

**Fig. 5:**
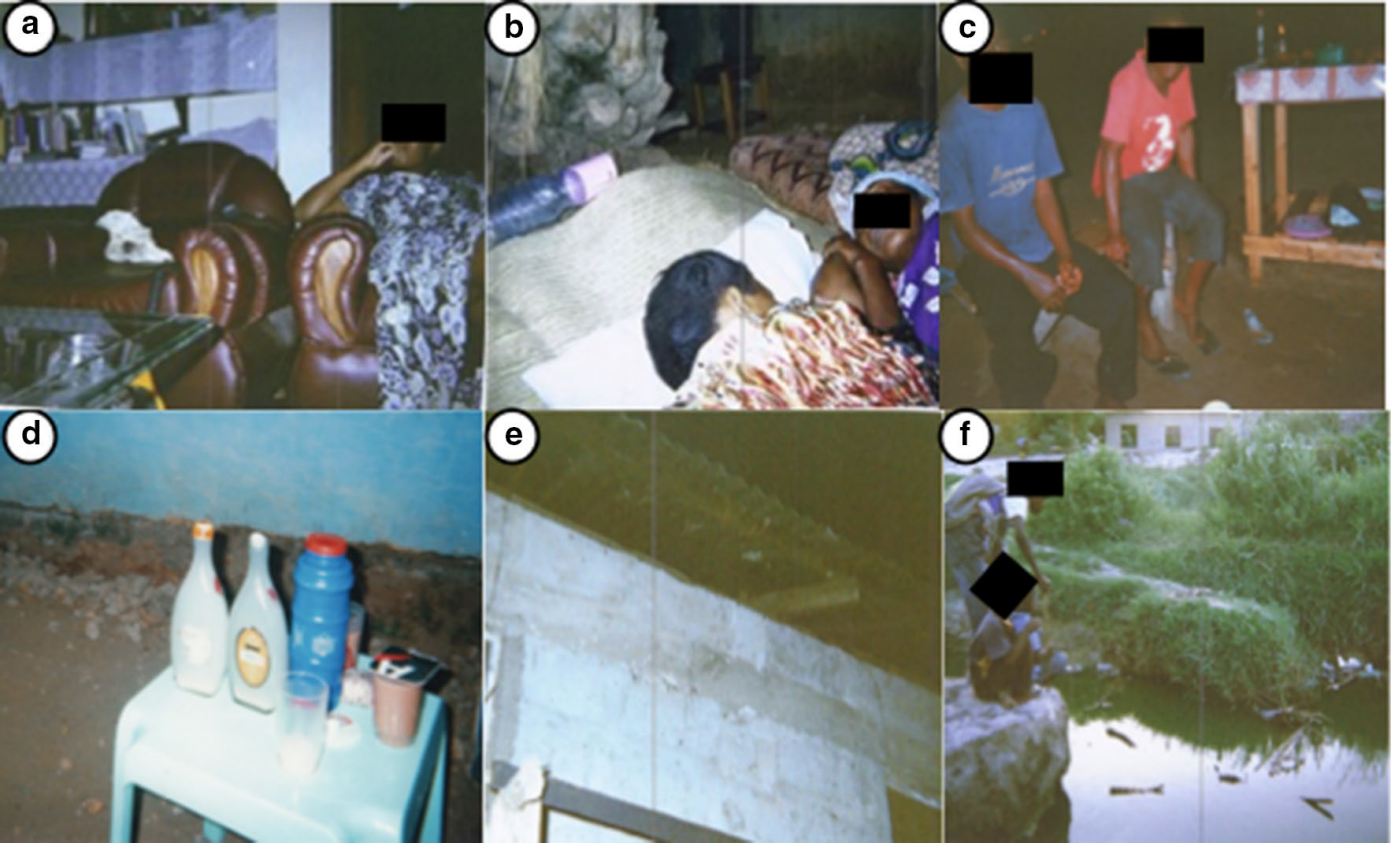
Photographs taken by community participants of perceived common malaria risk behaviours and activities. **a** Watching television in a sitting room before going to bed, **b** sleeping outdoors during funeral ceremonies, **c** chatting outdoors at night, **d** drinking outdoors at night, **e** living in a house with open eaves, **f** fishing activities

There was almost universal agreement among participants in all locations that there were currently few effective options for personal protection against outdoor biting mosquitoes, other than slapping and covering up with clothing. Exposure to outdoor-biting mosquitoes was seen to be of particular concern during livelihood and leisure activities, such as fishing at water bodies, street food vending, watching television before retiring to bed and attending funeral ceremonies (Fig. 5).

*‘Let us think about people who drink alcohol like that photo [referring *Fig. 5d *]…some people who may drink up to 2.00 am, without being protected from mosquitoes bites. All these people are exposed to malaria, regardless of the fact that such person use bed net at home.’* (Female, FGD participant, peri urban, low income)

*‘You can only put on* kangas *[clothing sheets] as protection against mosquitoes, or you can use your hands to slap them! There is no protection [outside a bed net]. If you don’t have trousers, there is nothing you can do.’* (Female, FGD participant, peri-urban low income)

Protective repellent products, such as topical skin repellents and repellent mosquito coils were reported by some participants across all urban settings as being used on specific occasions, such as in ceremonies, or while frequenting recreational drinking venues, and in business venues. In the rural setting, lighting a fire was also was mentioned by a few participants as a means to protect themselves outdoors. All these methods used in the outdoor environment were perceived to be unsatisfactory or inadequate. Indeed it is notable that no photographs were taken of topical repellents or coils, so they do not feature in Fig. 4. While currently available measures for outdoor protection were seen as inadequate, there was a widely voiced view that the best method for protecting against outdoor biting would be through larval source management, through environmental management and larviciding implemented by the government rather than by individual householders.

*‘It is true that they normally educate us on cleanliness as the way of preventing mosquitoes but I think after cleanliness, the important thing here is to have a program of applying insecticide in places where mosquito breed, from time to time to kill them. Surely for me, the only thing the government should do is to find insecticides to kill mosquitoes in their breeding places.’* (Female, FGD participant, peri-urban, low income)

This view was perhaps influenced by memories of previous intervention efforts; some participants referred to historical mosquito abatement programmes, particularly that implemented as a pilot evaluation in Dar es Salaam and Tanga in the 1980s [42]:

*‘We need to keep our environment clean, and the government should find an alternative way to help us. I remember in 1980, we didn’t use bed nets for like 5* *years, mosquitoes were not problem. There were a certain trial project that used to fumigate houses and trees, and also treat puddles. For all 5* *years, there were no mosquitoes. That project were conducted in Tanga and Dar es Salaam.’* (Female, PVGD, peri urban, low income)

## Factors influencing use of personal protection measures against mosquito bites

**Fig. 6:**
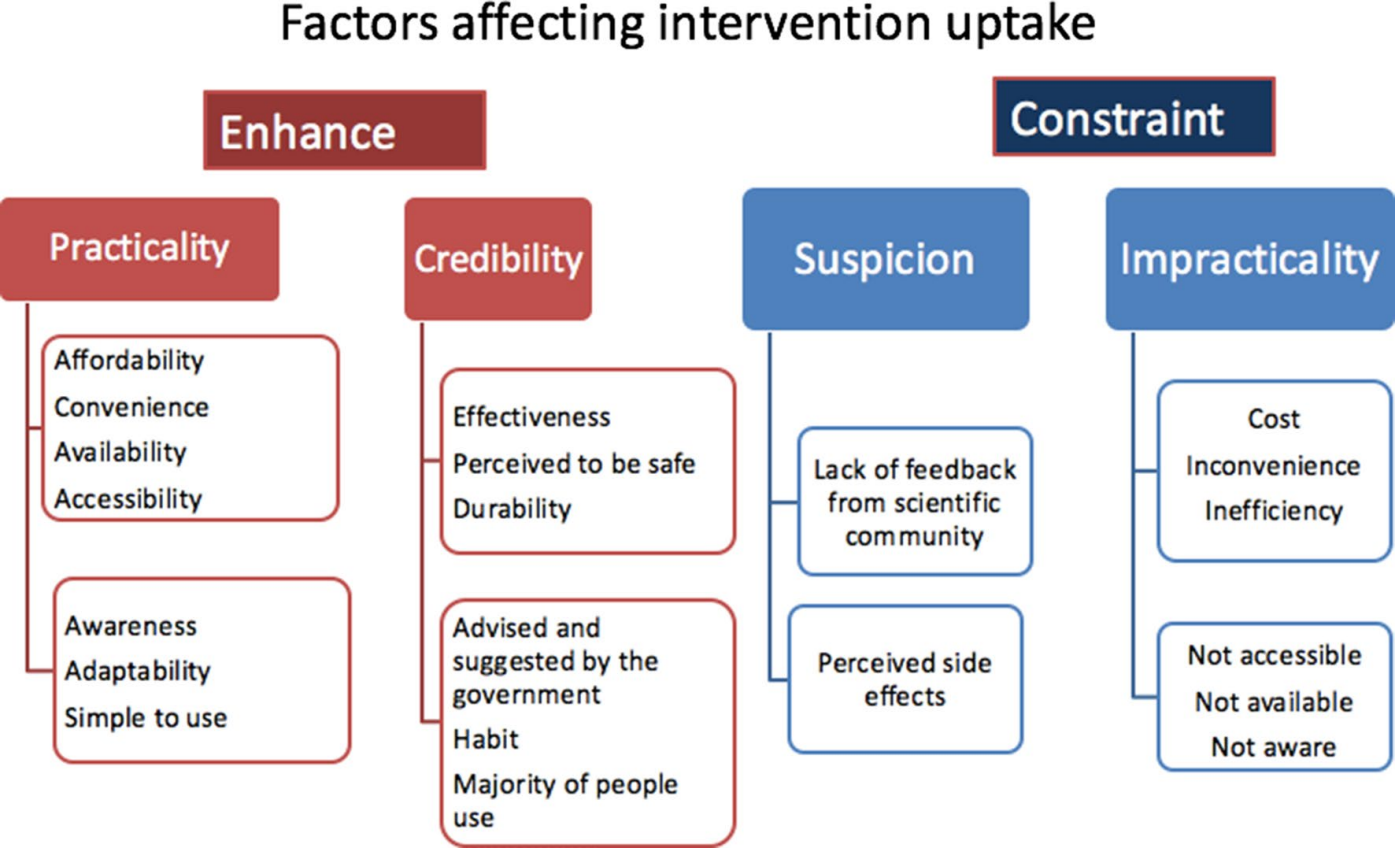
A schematic outline of factors affecting intervention uptake

Several factors were reported by participants to be important in guiding the use of mosquito protection measures. These can be categorized into factors that enhance use and those that constraint use (Fig. 6). The two keys factors enhancing use were: practicality, which incorporates affordability, convenience, availability, adaptability, and simplicity of use, and credibility which involves effectiveness, perceptions of safety, durability, endorsement by the government, habit, awareness and majority of use. The key factors constraining use were: suspicion, which arises from perceptions of potential side effects and lack of feedback/endorsement from the Government or the scientific community, and impracticality relating to cost, inconvenience, inefficiency, lack of availability, accessibility or awareness.

Affordability was by far the most frequently reported factor enabling or constraining the uptake and use of protection measures against mosquitoes. For example, the majority of participants from the low-income locations attributed their high reliance on LLINs to these being the least expensive method, as well as convenient and readily available. Another frequently cited advantage reported to contribute to long-term affordability was the durability of LLINs, which can be easily repaired.

*‘The price of [topical] repellent is about 1500 shillings* (equivalent to US$0.75) *per tube, so how many times can I and the whole family apply it? That’s why we are saying bed nets help us more, because they last longer. You cannot use it for one or 2* *days only*—*you just need to repair them.’* (Female, FGD participant, urban low income)

However, the durability of LLINs from a specific source, most notably those that were provided free of charge during national distribution programmes [30, 43], were frequently questioned in all study locations. Participants reported that the holes in these free polyethylene nets became enlarged after being washed, and some also remarked that they had relatively big holes to begin with.

*‘You know these current distributed nets (bed nets) have been made by plastics and they have big holes so mosquitoes can penetrate inside the net.’* (Female, FDG participants, urban, low income)

Topical repellents and insecticide sprays were frequently mentioned by the majority of participants from low-income level as being too expensive.

*‘Can you take 2000 shillings* (equivalent to US$0.90) *to buy spray while you don’t have food? Life is very difficult and 2000 shillings is a lot for poor people. We can’t afford*-*we have children to take care of.’* (Male, FGD participants, urban, low income)

Mosquito-proofing houses was considered expensive by the majority of participants from low-income urban and rural locations. These participants frequently reported that their houses had no window screens or ceiling boards, allowing mosquitoes easy entry into their houses. Many of the participants from these locations indicated they would like to use such mosquito-proofing measures if they could afford them.

Effectiveness was also mentioned by people from all locations as a factor which influences the uptake of mosquito protection measures. LLINs were generally reported to be the most effective protection method, as well as the most affordable. While insecticide sprays were appreciated for their immediate effectiveness by users, they were also criticized in equal measure for their lack of any residual effect, necessitating prohibitively expensive daily reapplication. By contrast, mosquito coils were perceived to be a more affordable option than topical skin repellents and insecticide sprays, but were perceived by some participants as ineffective.

The availability of protection measures, and indeed awareness of their existence, also emerged as factors which influence the use of a tool. In the rural study location, LLINs were perceived to be the most readily available tool and almost all participants from this location cited LLINs as the only known tool available for protection against mosquitoes.

*‘We use bed nets and we do not know other tools. There is not any other tool in our village.’* (Female, FGD participant, rural, low income)

Social factors, such as habit, familiarity, and norms of use, as well as government recommendation, also emerged as important drivers of awareness, acceptance and uptake. The majority of participants from low-income locations said that LLIN use had become the social norm and that their use of LLINs had been encouraged by seeing them in widespread use, and their own experiences over a long period of use in their households:

*‘I use bed nets because I have known them since I was very young. Of course this is what my parents used to do. They used it as an effective way to protect against mosquito bites*.*’* (Female, IDI participant, rural low income)

On the other hand, suspicion of new products, about which little was known and/or few had experienced, emerged as a major constraint to their use. This was a theme that cut across gender, age and income class. For example, across all study locations, the majority of participants perceived repellent formulations for topical application to the skin as causing influenza-like symptoms and numbness, and even having potential negative effects on human reproductive health, including causing breast development among men. In all discussions regarding the use of skin repellents, concerns about side effects outweighed the perceived potential benefits. Children were perceived to be more vulnerable to possible side effects of topical repellents than adults and, throughout the study, only a few adults reported using them even occasionally.

*‘Many words have been spoken against the use of [topical] repellents. Some people say they can cause numbness, and others say they can have negative effects on the reproductive system.’* (Male, FGD participant, peri-urban, low income)

Suspicion of a protective tool was not, however, restricted to new products. A small but notable number of participants were suspicious of LLINs, in particular those which were provided free of charge by the Government, despite LLINs having been in widespread use in Dar es Salaam for over 20 years. Some male participants expressed concern that the insecticide used may harm their virility.

*‘People are saying a lot concerning the free bed nets, they say it has an insecticide which reduce men’s ability in sexual activity.’* (Female, FGD participant, urban, low income)

*‘I never use a bed net which was provided freely by the local government office. I heard that it has insecticide which reduces men’s ability in sexual activity. I would rather buy a bed net in the shop than using the government bed nets.’* (Male, FGD participant, peri-urban, income)

A more widely expressed, broader concern was that if the insecticide can kill or repel mosquitoes, what effects will it have upon humans?

*‘Nowadays bed nets are treated with insecticide which kills mosquitoes instantly when they touch the bed net, I wonder what is its effect upon a human being who is sleeping under it for years? I think they should tell us how harmful it is to humans. Even for very small effects, we must be informed, eeeh!*’ (Female, IDI respondent, urban rich)

Despite such perceptions of potential risks, the majority of participants nevertheless said that they used LLINs, and only one participant from the peri-urban location reported not using a LLIN specifically because of these concerns.

Mosquito coils were also suspected by a few participants to cause negative side effects, including influenza-like symptoms that have been documented elsewhere [44], with one participant concerned about the linkage with premature greying of hair.

*‘Coils are not efficient at all, and you can fall asleep immediately after using it. Some people also said that, if used frequently, it can change your hair colour to grey.’* (Male, FGD participant, urban low income)

Impracticality was also considered to constrain the selection of protection measures. For the majority of participants, except those from the relatively wealthy urban location, insecticide sprays were perceived as an “impossible tool” in houses without screened windows, and with large eaves gaps between the roof and walls. In addition, the effectiveness of mosquito-proofing houses was said to depend on making sure that doors and windows are closed to prevent mosquitoes from entering, which was considered difficult for families with many household members.

## Discussion

The need for the development of novel strategies for vector control to enhance progress towards eliminating malaria transmission is widely recognized. There is also broad agreement that to maximize effectiveness, new tools and strategies need to take account of the context within which they will be implemented. This study used a combination of qualitative and participatory methods to explore: perceptions of mosquitoes, vector control tools employed, and the factors influencing the uptake and use of these tools among householders across a range of socio-economic and environmental contexts in Dar es Salaam, Tanzania.

Participants in this study complained that mosquitoes were a widespread and growing problem in Dar es Salaam and, in common with many others studies in Tanzania and elsewhere in Africa over the past 25 years, the major concerns relating to mosquitoes were nuisance biting and mosquito-borne diseases, the most prominent of which was malaria [45–51]. The pictures taken by the PV participants in the current study, and endorsed by participants in the community FGDs, show that that dirty stagnant water, rubbish and grasses are considered to be important sources of mosquitoes. This finding has been commonly reported in many malaria-endemic countries [47, 52–54] but in the current study the PV participants also used the pictures they had taken to demonstrate the difference between the wet places where the mosquitoes breed and the dark, predominantley dry places where they hide. Dark places inside houses were specifically identified as hiding or resting sites, a finding also reported in a study in Ethiopia [53]. In common with the findings of a study undertaken in Dar es Salaam 25 years ago [47] and other studies from endemic areas of Africa, there was much lower recognition that a particular type of mosquitoes might be responsible for malaria transmission, or that different types of mosquitoes might have different habitats for breeding. This is perhaps not surprising in light of most of the vector control activities and health education messaging that has been implemented in Tanzania over the past century, and more recently during mass distribution of free LLINs in which the focus has been on generally creating a ‘clean’ environment [30, 43, 55]. A recent ethnographic study of the Urban Malaria Control Programme (UMCP) in Dar es Salaam reported how these historic vector control activities are still recounted by current UMCP personnel and the clear memories elderly residents have of taking part in public health clean-up campaigns to remove potential mosquito breeding sites [56]. The focus of many of these campaigns has been on general environmental cleanliness rather than the specifics of reducing potential breeding sites for any particular species of mosquito. For example, the Government’s *Mtu ni Afya* (A Person is Health), a mass behaviour change communication (BCC) campaign in the 1970s aimed at improving the health of rural populations, focussed on widespread high-burden diseases, including malaria, and frequently stressed the importance of general environmental cleanliness as a means of sustainable, community-based malaria control [55]. *Mtu ni Afya*, and many public health messaging campaigns since then, emphasized cutting down grasses and other tall vegetation around houses, and removing obvious bodies of stagnant water as methods for vector control. While these recommendations may have other health benefits, clearing grasses and bushes is thought to have little impact on malaria transmission by African vectors [57]. Some of categories of the mosquito-breeding sites mentioned in these campaigns were suitable for *Anopheles* but often participants named sites that were unimportant for malaria vectors but suitable for other numerous vectors of neglected tropical diseases, especially *Culex* spp. Furthermore, their emphasis on *stagnant* water, meaning water that does not flow is misleading with regard to the quite specific general properties of malaria vector breeding sites, because for many people this term implies dirty water. For malaria campaigns, more accurate, informative and practically actionable messaging is urgently needed about *Anopheles* larval ecology.

As summarized in the classic monograph describing the biology of *Anopheles gambiae* [58]: ‘*The water in open pools used for breeding may be clear or muddy*.’ But: ‘*It is also well known that gross pollution of either vegetable or animal origin is usually inimical to the species*.’

In the experience of the authors, the simplest rule of thumb for lay persons to identify potential malaria vector habitats in Africa is that these mosquitoes can breed in any body of water, which is either still or has sheltered fringes with little if any flow, and contains water that is sufficiently uncontaminated with organic matter for livestock to drink it [58]. With some rare exceptions, water storage containers and water bodies lacking regular exposure to direct sunlight are rarely used as breeding sites by African malaria vectors: these are more likely to produce day-biting *Aedes* that cause dengue, chikungunya and zika. Furthermore, malaria-carrying *Anopheles* do not breed in water bodies that are heavily contaminated with organic matter, such as pit latrines, soakage pits or sewers, even if they are exposed to direct sunlight: these are far more likely to produce culicines, *Culex quinquefasciatus* in particular, which commonly transmit lymphatic filariasis [58].

Perhaps unsurprisingly in view of the norms of vector control practice that have been implemented through urban vector control activities spanning more than a century in Tanzania [47, 56], the majority of participants in this study stressed the importance of environmental management and larvicide application to mosquito-breeding sites as the most effective strategies for controlling outdoor-biting mosquitoes and malaria. Such views are consistent with entomological evidence that larval source management (LSM) is an appropriate intervention wherever feasible, because it prevents the emergence of adult mosquitoes at source, and is particularly useful for species that are otherwise difficult to kill because they exhibit various forms of behavioural evasiveness [59–61]. During the colonial era LSM in Dar es Salaam was the responsibility of local authorities and enforced through regulation [56]; today the majority of participants in this study perceived that LSM activities should be the collective responsibility between community members and local governments. Achieving successful LSM in democratic regimes needs four elements: political will and commitment, community sensitization and participation [62].

Consistent with the findings from many other studies [5, 45, 46, 63, 64] including the study undertaken in Dar es Salaam and Tanga during the early 1990s [47], participants across all the study locations reported employing some form of protection against mosquito bites. However, while burning repellents such as mosquito coils was the method most frequently mentioned as being used to protect against mosquitoes in the study undertaken in Dar es Salaam and Tanga in the early 1990s [47], by the time of this current study LLINs were the most frequently mentioned protection method. Interestingly, the authors of the earlier study report that participants recognized the effectiveness of LLINs but the main constraint to their use was the cost [47]. By contrast, in the current study the participants on low income suggested that LLINs were “part of culture”, consistently mentioned as the first-choice malaria prevention measure due to their affordability, effectiveness, convenience of use, and ready availability, especially in low-income areas. The transition of LLINs from a luxury good to their use as a social norm and part of the culture is likely to reflect the cumulative impact of more than 20 years of subsidized, and subsequently free, net distribution and associated BCC campaigns in Tanzania [30, 43].

The findings in this study on the importance of effectiveness, affordability, availability, and convenience of use, on the uptake of an intervention are similar to those of other studies in Tanzania [46, 63, 64]. Social factors such as recommendations from the Government (if the Government is trusted as a source of accurate information) and internalization through habitual use and social norms have been noted as motivation factors for use of measures for protection against mosquitoes by other studies in Tanzania and Mozambique [45, 63, 65]. However, for most participants in the study presented here, LLINs alone are not sufficient to fully address the challenges of malaria exposure and nuisance biting, and this view is consistent with the observations of others in Tanzania [63] and elsewhere in Africa [66].

Mosquito-proofed housing was mentioned frequently, but not as frequently as LLINs even though window screening in particular has achieved high coverage in recent years, particularly in the wealthier areas of the city [5, 31]. This may reflect greater consciousness of the widely promoted, singular role of LLINs for protection against mosquitoes and malaria in deliberate BCC campaigns, whereas housing modifications such as window screening and ceilings have multiple functions other than prevention of mosquito entry and have never been actively subsidized [5, 22, 31]. Despite the effectiveness of improved housing as malaria vector control method [67–69], it has received inadequate attention from funders and policy makers [69]. Perhaps what is required is further studies, including to establish the cost-effectiveness of the house proofing per case averted in different malaria transmission settings. Also, by identifying and validating the most practical and effective means of improving houses, with potential of subsidies of such means for households.

Outdoor exposure to mosquito bites in the evenings and early mornings has been reported as a cause of residual malaria transmission in many African settings [4, 70–73] including Dar es Salaam [5, 22, 23]. The community perceptions reported here are consistent with combined quantitative entomological and social science surveys demonstrating that, even in parts of Africa with vectors exhibiting classically nocturnal biting behaviour [74], once residents are protected by LLINs, approximately half of their remaining biting exposure occurs outdoors, where no satisfactory personal protection method is currently available. In the current study, slapping and covering up with clothing were reported as the most common method for protecting against outdoor biting. Studies from Kenya and other countries indicate that insecticide-treated clothing (*shukas*, *diras, chaddar, saris, jalbaab*s, *ma’awis*, and shirts) and bedclothes (sheets and blankets) are protective against malaria [75–78]. Insecticide-treated personal clothes may, therefore, provide useful options for protecting against outdoor biting in this setting, where high body surface coverage with clothing is a cultural norm amongst many residents. Nevertheless, considerable variation in clothing practices exists amongst residents of Dar es Salaam and elsewhere in Africa, so alternative personal protection measures will be required, the most obvious of which are repellents. The view of the participants in this study was that the need for frequent re-application make topical repellents too expensive for routine use. Moreover, none of the currently available topical repellents or mosquito coils fulfil the clinical epidemiological requirements for recommendation as malaria control applications [79]. However, emerging prototypes of a low-cost, low-technology emanator that releases protective repellent vapour for months at a time [31, 80–83] look promising as a malaria control intervention and merit further evaluation. If such prototypes prove to be effective, it is likely that on their introduction they would still face some hostility and suspicion. Ambivalence towards new public health interventions has a long history in Africa [84, 85]. Inadequate information, fear of side effects, lack of evidence of effectiveness and impracticality of use, all contribute to scepticism and concerns when new tools are offered [44, 45, 63, 64, 86–89]. In addition, when new products are first introduced, cost and availability are often major constraint to their widespread adoption [46, 47, 64, 90, 91]. Even among interventions such as LLINs that have become widely accepted and used, suspicions about the potential effects of the insecticides can remain [63, 64, 86, 92, 93]. In this study it is encouraging that, despite residual fears expressed by a few participants, the use of LLINs has become a social norm. This suggests that given time, effective vector control tools, promoted by trusted sources and made widely available, affordable and accessible can become ‘part of the culture’.

## Conclusions

This study successfully combined conventional FGD and IDI methodology with the novel PV methodology, to involve communities in documenting the problems they experience with respect to protecting themselves against mosquitoes. The results obtained indicate strong community support for traditional LSM approaches targeting both malaria vectors and nuisance-biting mosquitoes. Under current democratic regimes such strategies require the involvement of both communities and local government, and mostly important political will to help effective implementation. New methods for personal protection outdoors are also needed, as existing options are perceived to have considerable limitations and risks. Insecticide-treated clothing and long-lasting delivery formats for vapour-phase insecticides and repellents should be developed and evaluated for programmatic use. Affordability, availability, effectiveness, and habit appeared as key factors influencing the uptake of mosquito control measures. However, even when these criteria are satisfied, new methods may require time and user experience to achieve correspondingly positive reputations and trustworthiness.

## Additional files

**Additional file 1.** Semi-structured discussion guide for *photovoice* interviews on perceptions and relevance of the photographs in relations to mosquitoes.

**Additional file 2.** Semi-structured discussion guide for IDIs, FGDs and PVGD.

**Additional file 3.** Informed consent for photovoice participants (1) English version, (2) Swahili version.

**Additional file 4.** Informed consent for IDIs participants (1) English version, (2) Swahili version.

**Additional file 5.** Informed consent for FGDs participants (1), English version (2) Swahili version.

## Abbreviations

ACT: artemisinin-based combination therapy
BCC: behaviour change communication
FGD: focus group discussion
IDI: in-depth interview
IRS: indoor residual spraying
LSM: larval source management
LLIN: long-lasting insecticidal net
SSA: sub-Saharan Africa
RDT: rapid diagnostic test
PVGD: photovoice group discussion
PV: photovoice
TCU: ten cell units
