# Supplementary material for: Informing new or improved vector control tools for reducing the malaria burden in Tanzania: a qualitative exploration of perceptions of mosquitoes and methods for their control among the residents of Dar es Salaam
Source: Malar J. 2017 Oct 11;16:410. doi: 10.1186/s12936-017-2056-9 (PMC5637339; doi:10.1186/s12936-017-2056-9)
Supplement: Supplementary file 1 — Additional file 1. Semi-structured discussion guide for photovoice interviews on perceptions and relevance of the photographs in relations to mosquitoes. [file 12936_2017_2056_MOESM1_ESM.doc]

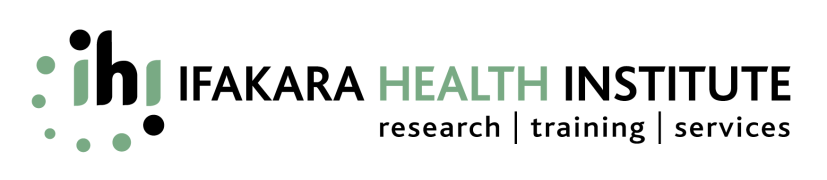


**PHOTO VOICE INTERVIEW GUIDE**

**1.** Show the participants their photos

2. Ask participants to select 10 most relevant photos

3. Ask them to describe what the photos show

4. Ask them to describe what the photos mean

5. Ask them to explain their relevance

6. Engage the study participant in general discussion about mosquitoes and prevention

***Note: refer to the interview and FGD topic guide for general questions***

**MWONGOZO WA MASWALI MAHOJIANO YA PICHA SAUTI**

1. Waonyeshe washiriki picha zao
2. Waambie washiriki wachague picha 10 muhimu zaidi kuhusu Mbu
3. Waulize kile kinachoonyesha kwenye picha kinaelezea nini
4. Waelezee picha zina maana gani
5. Watoe maelezo ya umuhimu wa picha
6. Anza majadiliano ya jumla kuhusu mbu na namna ya kujikinga

*Kumbuka: Tumia mwongozo wa maswali ya mahojiano ya vikundi kwa maswali ya jumla.*
