# Supplementary material for: Informing new or improved vector control tools for reducing the malaria burden in Tanzania: a qualitative exploration of perceptions of mosquitoes and methods for their control among the residents of Dar es Salaam
Source: Malar J. 2017 Oct 11;16:410. doi: 10.1186/s12936-017-2056-9 (PMC5637339; doi:10.1186/s12936-017-2056-9)
Supplement: Supplementary file 2 — Additional file 2. Semi-structured discussion guide for IDIs, FGDs and PVGD. [file 12936_2017_2056_MOESM2_ESM.docx]

**
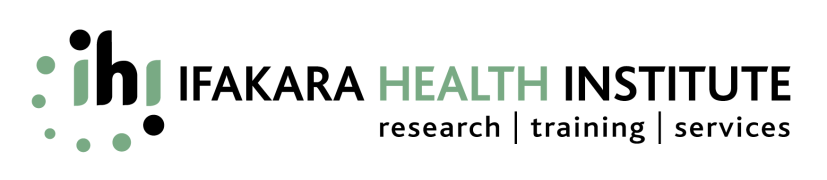
**

**TOPIC GUIDE FOR INTERVIEWS, FOCUS GROUP DISCUSSIONS AND PHOTOVOICE**

Household behaviour in relation to mosquitoes

1. Perceptions of mosquitoes

a. Perceptions of where mosquitoes come from

b. Problems or benefits associated with mosquitoes

i. **Probe** why mosquitoes are a problem or benefit

ii. ***Probe*** when and where mosquitoes are a problem or benefit

iii. Anyone who is more at risk of mosquito problems

2. Introduce pictures/photos and ask participants if they associate any of them with mosquitoes and

they should provide/discuss reasons

3. Actions taken to avoid or prevent mosquitoes

a. Probe for tools used inside and outside the home and reasons for use

b. Probe on the source of the tools

c. Probe for differences in actions taken by age or gender

d. Probe for reasons for any difference by age or gender

e. Probe inside and outdoor differences

4. Perceptions of any changes of mosquito density over the few year?

a. Probe for factors that might have led to this pattern

**MWONGOZO WA MASWALI KWA WASHIRIKI YA MAHOJIANO YA VIKUNDI NA MTU MMOJA MMOJA**

*Tabia za kaya dhidi ya mbu*

1. *Mtazamo juu ya Mbu*

a. Dadisi mtazamo wa Mbu wanatoka wapi?

b. Matatizo au faida zinazoletwa na Mbu

i. Dadisi; Kwanini mbu ni tatizo au ni faida?

ii. Dadisi; Wakati gani na mahali gani Mbu ni tatizo au faida

iii. Je ni nani aliye katika hatari zaidi kwa matatizo yanayosababishwa na Mbu

*2.. Onyesha picha na uliza washiriki au mshiriki kama wanazihusisha na Mbu, Kama wanazihusisha na Mbu watoe sababu na wazijaadili sababu hizo.*

*3. Matendo yanayofanyika au zana zinazotumikai kujikinga au kuzuia au Mbu*

a. Dadisi mbinu au zana zinazotumika kujikinga au kuzuia Mbu nje na ndani ya nyumba na sababu zinazopelekea matumizi hayo

b. Dadisi zana hizo zinapopatikana

c. Dadisi kama kuna tofauti ya zana za kujikinga kwa kulingana na tofauti ya umri au jinsia

d. Dadisi sababu ya tofauti hiyo kulingana na umri au jinsia

e. Dadisi sababu ya matumizi tofauti ndani na nje ya nyumba

4. Dadisi mtazamo wa mabadiliko ya kuongezeka au kupungua kwa mbu katika eneo lao

a. Dadisi sababu za mabadiliko hayo
