# Supplementary material for: Informing new or improved vector control tools for reducing the malaria burden in Tanzania: a qualitative exploration of perceptions of mosquitoes and methods for their control among the residents of Dar es Salaam
Source: Malar J. 2017 Oct 11;16:410. doi: 10.1186/s12936-017-2056-9 (PMC5637339; doi:10.1186/s12936-017-2056-9)
Supplement: Supplementary file 3 — Additional file 3. Informed consent for photovoice participants (1) English version, (2) Swahili version. [file 12936_2017_2056_MOESM3_ESM.zip › Informed consent for Photovoice-Swahili version.doc]

**Jina la Mradi:** Mbinu Mpya za Kudhibiti Wadudu Waenezao Magonjwa Afrika (African Vector Control : New Tools AvecNet ).

**Lengo:** Kuboresha mbinu zilizopo au kupata mbinu mpya za kudhibiti mbu ili kupunguza tatizo la malaria katika bara la Afrika

Taarifa ya mradi na ridhaa kwa washiriki wa utafiti wa picha-sauti

| **Taasisi** | **Mhusika** |
| --- | --- |
| Taasisi ya afya Ifakara | Christina Makungu |

**Utafiti huu unahusu nini?**

Madhumuni ya utafiti ni kuboresha au kupata mbinu mpya za kudhibiti mbu ili kupunguza maambukizi ya malaria. Kwa ujumla tunataka kujua mtazamo wako juu ya mbu na njia zinazotumiwa katika kaya hii ili kujikinga na mbu na sababu zinazopelekea kutumia njia hizo. Uzoefu, mtazamo na maoni yako hili yatasaidia kupata taarifa zitakazo tuwezesha kupata mbinu mpya au kuboresha mbinu zilizopo ili kupunguza malaria kwa kudhibiti mbu.

**Nini kitatokea:** Tutawapatia kamera isiyoya kudumu kwa idadi ndogo ya watu katika eneo hili ili wapige picha ambazo wanadhani zina uhusiano. Kabla ya kutumia kamera hizo, tutawafundusha washiriki namna ya kutumia hizo kamera kwa kuzingatia na kuheshimu haki na usalama wa watu wengine. Pia tutafanya mahojiano na mtu mmoja mmoja na kwenye vikundi katika eneo hili kuhusiana na mada hii.

**Kazi utayofanya na kamera tutakayokupa:** Kama nilivyokueleza hapo, tutakupatia kamera hii ambayo utaitumia kupiga picha ya mambo unayoyahusisha na mbu. Kama uko tayari kushiriki utafiti huu, tutakuomba uhudhurie mafunzo yatayohusu upigaji picha, namna kamera inavyofanya kazi na jinsi ya kuitumia kwa kuheshimu na haki na usalama wa watu.Tutakupatia kamera hii kwa wiki moja au mbili ili upige picha, kisha tutazitoa picha katika kopi maalumu na kukuomba ushiriki katika mjadala wa picha zitakazoletwa. Ukikubali kushiriki, maongezi hayo yatarekodiwa katika kanda ama kisahani (disc) lakini rekodi hizi zitaharibiwa mwishoni mwa mradi. Kama hutapenda majadiliano kurekodiwa au kusitisha rekodi, mtafiti atafurahi kutekeleza ombi lenu. Mwishoni mwa utafiti, utapewa taarifa rahisi na fupi ya mradi na matokeo ya utafiti.

**Madhara :** Hakuna madhara yoyote katika kushiriki utafiti huu.

**Faida :** Hakuna faida binafsi utakayopata kushiriki katika utafiti huu ila utapatiwa kamera ya ziada kwa matumizi yako binafsi. Vilevile matokeo ya utafiti huu yatatusaidia kupata taarifa zitakazotuwezesha kudhibiti maambukizi ya malaria kwa kuboresha mbinu zilizopo au kupata mbinu mpya zinazokubalika katika jamii.

**Usiri:** Picha na maelezo utakayotupatia yatahifadhiwa kwa usiri kwa ajili ya mradi tu. Maelezo na picha yatahifadhiwa katika chumba salama kinachofungwa. Pia yatahifadhiwa katika kompyuta inayolindwa kwa neno la siri. Utaweza kuona maelezo au picha ulizotupatia wakati wowote ukitoa taarifa yenye kueleza sababu za msingi za kutaka kufanya hivyo. Jina lako halitatumika kutambulisha maelezo yoyote uliyotupatia katika ripoti ama maandishi yoyote ya mradi. Hakuna picha za kumtambulisha mtu au picha zenye kutambulisha mtu zitakazotumiwa bila kuchukua ridhaa ya maandishi ya mhusika kutoka kwa aliyechukua picha na aliyepigwa picha.

Taarifa zitakazopatikana katika utafiti huu zitatumika kwa ajili ya mradi tu , bila kumtambulisha mtu binafsi. Kwa ridhaa mshiriki, majina bandia yatatumika kuelezea jambo fulani lakini hakutakuwa na maelezo yoyote yatayotolewa kumtambulisha mhusika.

**Kujitoa ushiriki:** Utakuwa huru kujitoa katika utafiti wakati wowote bila kulazimika kutoa maelezo ya kuamua kufanya hivyo na bila kupigwa faini yoyote kwa kufanya hivyo. Unaweza kujitoa kwa kuongea na mhusika wa mradi ama kwa maandishi.

**Nini kinachofuata**: Ushiriki wako katika utafiti huu ni wa hiari. Ukikubali kushiriki utaombwa kusaini fomu ya ridhaa. Na iwapo utataka kusitisha ushiriki wako katika utafiti huu unaombwa kutoa tarifa ya maneno au maandishi kwa watafiti na hakuna athari yoyote itakayokupata.

**Maswali:** Kama una maswali yoyote tafadhali kuwa huru kumuuliza mhusika yeyote wa utafiti.

**Anuani ya mtafiti/mhusika:** Christina Makungu

S.L.P 78373

Mikocheni, Kiko avenue

**Dar-es-salaam**

**Namba ya simu ya mkononi: 0688-423982**

**Fomu ya Ridhaa ya pichasauti**

Mimi, ________________________________ ninakubaliana na maelezo yanayohusu utafiti. Nimesoma au nimesomewa maelezo ya utafiti, nimeelewa na kuridhika kabisa. Sababu za kupewa kamera nimeelezwa na pia nimeelewa kuwa picha nitakazopiga zitatumiwa kwa utafiti, kwa hiyo zinaweza kutumiwa katika machapisho na ripoti. Nimefundishwa pia namna ya kupiga picha vizuri bila kuathiri haki na usalama wa mtu au watu katika jamii hii. Ninatambua kuwa ninahaki ya kujitoa kwenye ushiriki muda wowote bila kuathiri matokeo ya utafiti huu.

**Tafadhali, Unaombwa kujaza fomu hii..**

**Ninakubali kushiriki katika utafiti huu** *tafadhali weka alama ya vema katika kisanduku kimojawapo* ** Ndiyo  Hapana**

**Ninakubali kujadili mada ya picha zilizopigwa na:** *tafadhali weka alama ya vema katika kisanduku* *kimojawapo*

** Ndiyo	wapigaji picha wengine pamoja na mtafiti**

** Ndiyo	mtafiti peke yake**

**Ninakubali kutumiwa kwa nukuu zitakazotokana na mjadala wa utafiti huu kutolewa kwenye machapisho,** *tafadhali weka alama ya vema katika kisanduku kimojawapo*

** Ndiyo**

** Ndiyo , ila maelezo yatakayonitaja mimi, mwajiri wangu na cheo changu yatafichwa**

** Hapana, ila maelezo nitakayoyatoa yajumuishwe**

**Sahihi ya Mshiriki**: __________________________

**Tarehe** _______________________

(JINA LA MSHIRIKI KWA HERUFI KUBWA)__________________________________

**Sahihi au dole gumba la shahidi: _____________________________**

**Tarehe __________**

(TAFADHALI ANDIKA JINA KWA HERUFI KUBWA**)_________________________**

Ninathibitisha kuwa nimefuata taratibu zote kama inavyopasa kupata ridhaa ya mashiriki.**Sahihi ya Mtafiti:** ____________________________ **Tarehe** ____________

**Jina la Mtafiti:** _________________________ (Tafadhali andika)
