# Supplementary material for: Informing new or improved vector control tools for reducing the malaria burden in Tanzania: a qualitative exploration of perceptions of mosquitoes and methods for their control among the residents of Dar es Salaam
Source: Malar J. 2017 Oct 11;16:410. doi: 10.1186/s12936-017-2056-9 (PMC5637339; doi:10.1186/s12936-017-2056-9)
Supplement: Supplementary file 3 — Additional file 3. Informed consent for photovoice participants (1) English version, (2) Swahili version. [file 12936_2017_2056_MOESM3_ESM.zip › Informed concent photovoice-English version.doc]

**AvecNet project: New or improved vector control tools for reducing malaria burden in Africa**

**Informed consent for Photovoice participants**

| **Institution** | **Investigator** |
| --- | --- |
| Ifakara Health Institute | Christina Makungu |

**Purpose of the research**

In this research, we want to develop better ways for controlling mosquitoes to help prevent the transmission of malaria. More specifically, we want to know your perceptions on mosquitoes, what householders do to protect themselves against mosquitoes and factors that influence their use. These views and experiences are important as they can provide information that can help to identify new tools and strategies for controlling mosquitoes.

**How will the information be collected?**

If you agree to participate, we will be giving disposal cameras to a small number of people in this community so that they can take photographs of things that they associate with mosquitoes. Before they use them, the people who are given the cameras will take part in a short training to learn about acceptable ways of using the camera to ensure that individual and community safety and rights are respected. We also plan to hold group discussions with you on your perceptions on mosquito and protection measure which are currently use against mosquito bites and factor which influence their use.

**Why do you want to give me a camera and what does it involve?**

We are providing you with this camera so that you can take photographs of things that you associate with mosquitoes. If you agree, then we will ask you to firstly take part in a short training about the photovoice method, how the camera works and about the ways of using the camera so that individual and community safety and rights are respected.

We will then give you a camera for a week or two so that you can take some pictures. When you have used the film we will develop the pictures for you and we will then ask you to participate in a discussion about the pictures, to describe why these ones were selected and what they show. We would like to tape-record the discussion to assist later in fully writing up the information.

If you are not comfortable with the discussion being recorded, or would like the tape to be switched off at any time, then the interviewer will be happy to comply with your request.

**Are there any advantages or disadvantages in taking part in this project?**

There are no individual benefits to taking part, but the information that we collect will help improve our understanding of the opportunities and challenges faced in the effective control of mosquitoes in this area.

**Who will have access to the information I give?**

We will not share individual information that is collected with anyone beyond a few people who are closely concerned with the research. All of our documents/recordings/photographs are stored securely in locked cabinets and on password protected computers. Any tapes from the recording of interviews and discussions will be destroyed as soon as they have been transcribed (usually within two weeks) and the transcripts will contain no information that will allow for the identification of individual participants. No photographs of identifiable individuals, or photographs that contain images that could be used to identify an individual will be used without first obtaining separate written consent from both the photographer and the individual in the photograph.

The knowledge gained from this research will be shared in summary form, without revealing individuals’ identities. With the agreement of participants, anonymous quotes may be used to illustrate general points, but if they are used they will not contain any information that will allow identification of individuals.

**What will happen if I refuse to participate?**

All participation in research is voluntary. You are free to decide if you want to take part or not. If you do agree you can change your mind at any time without any consequences.

**What if I have any questions?**

You are free to ask me any question about this research. If you have any further questions about the study, you are free to contact the research team using the contacts below:

**Physical contact of investigator**: Christina Makungu

P.O Box 78373

Dar es Salaam

Kiko Avenue, Mikocheni

**Mobile phone: 0688 423982**

**Consent Form for Photovoice participants**

I, ________________________________ have had the research explained to me. I have understood all that I have read (or have had read to me) and had my questions answered satisfactorily. The reasons for providing me with a camera have been fully explained and I understand that the photographs I take may be used for research purposes and may, therefore, be used as part of academic publications and reports. I have also received information on acceptable ways of using the camera and undertake to ensure that I respect individual and community safety and rights in the photos that I take. I understand that I can change my mind about taking part at any stage without any consequences.

**You will be given a copy of this signed consent form. Please complete the following:**

**I agree to take part in this research** *please tick one box*	** Yes  No**

**I agree to discuss the content of the photos with:** *please tick one box*

** Yes	Other photovoice participants and the researchers**

** Yes	The researchers only**

**I agree to the use of anonymous quotes that arise from these discussions in future publications,** *please tick one box*

** Yes**

** Yes , as long as *any* information that might be used to identify me, including the organization that employs me and my position within it remains anonymous**

** No, but the information I provide may be used to inform the overall analysis**

**Participant’s signature**: __________________________**Date** ______________________ (Please print name)

I certify that I have followed all the study specific procedures described in the SOP for obtaining informed consent.

**Designee/investigator’s signature:** ____________________________ **Date** ____________

**Designee/investigator’s name:** _______________________________**Time** ____________ (Please print name)
