# Supplementary material for: Informing new or improved vector control tools for reducing the malaria burden in Tanzania: a qualitative exploration of perceptions of mosquitoes and methods for their control among the residents of Dar es Salaam
Source: Malar J. 2017 Oct 11;16:410. doi: 10.1186/s12936-017-2056-9 (PMC5637339; doi:10.1186/s12936-017-2056-9)
Supplement: Supplementary file 4 — Additional file 4. Informed consent for IDIs participants (1) English version, (2) Swahili version. [file 12936_2017_2056_MOESM4_ESM.zip › Informed consent IDI -English version .doc]

**AvectNet Project: New or improved vector control tools for reducing malaria burden in Africa**

**Informed concent- in-depth Interviews**

| **Institution** | **Individuals** |
| --- | --- |
| Ifakara Health Institute | Christina Makungu |

**Purpose of the research**

In this research, we want to develop better ways for controlling mosquitoes to help prevent the transmission of malaria. More specifically, we want to know your views on the problems that mosquitoes can cause, what householders do to protect themselves against mosquitoes and factors that influence their use. Your views and experiences are important as they can provide information that can help us to identify new tools and strategies for controlling mosquitoes.

**What will happen:** If you agree to participate, we will arrange interview with you. The interview will last about 45 minutes to one hour. We would like to talk to you on whether you think mosquitoes are problem in this area or not, and the sort of problems that they cause and the methods you use in your household for mosquito control as well as the factors which influence their use. The interview will be recorded onto tape or disc but these recordings will be destroyed at the end of the project. If you are not comfortable with the interview being recorded, or would like the tape to be switched off at any time, the interviewer will be happy to comply with your request.

**Potential Risks:** There are no major risks to taking part in this study.

**Potential Benefits:** There are no individual benefits to taking part, but in answering our questions you will help us to design effective intervention that will be accepted and used by local communities to prevent mosquito-mediated transmission of malaria.

**Confidentiality:** We will not share individual information about you with anyone beyond a few people who are closely concerned with the research. All of our documents/recordings are stored securely in locked cabinets and on password protected computers. Tapes will be destroyed as soon as they have been transcribed (usually within two weeks) and the transcripts will contain no information that will allow for the identification of individual participants.

The knowledge gained from this research will be shared in summary form, without revealing individuals’ identities. With your agreement, anonymous quotes may be used to illustrate general points, but if they are used they will not contain any information that will allow identification of individuals.

**Withdrawal:** All participation in research is voluntary. You are free to decide if you want to take part or not. If you do agree you can change your mind at any time without any consequences.

**Payment:** There is no financial cost to you for participating in this study. You will not receive any payment for your participation.

**What happens next**: Your participation in this study is entirely voluntary. If you agree to participate, you will be asked to sign a consent form.

**What if I have any questions?**

You are free to ask me any question about this research. If you have any further questions about the study, you are free to contact the research team using the contacts below:

**Physical contact of investigator**: Christina Makungu

P.O Box 78373

Dar es Salaam

Kiko Avenue, Mikocheni

**Mobile phone: 0688 423982**

**Consent Form for In-depth Interview**

I, ________________________________ have had the research explained to me. I have understood all that has been read and had my questions answered satisfactorily. I understand that the data collected during this interview are to be used for research purposes and may, therefore, be used as part of academic publications and reports. I understand that I can change my mind about taking part at any stage, or refuse to answer any question, or ask for the interview to be terminated.

**You will be given a copy of this signed consent form.**

**Please complete the following:**

**I agree to take part in this interview** *please tick one box*	** Yes  No**

**I agree to have the interview tape recorded** *please tick one box* ** Yes  No**

**I agree to the use of anonymous quotes in future publications,** *please tick one box*

** Yes**

** Yes , as long as *any* information that might be used to identify me, including the organization that employs me and my position within it remains anonymous**

** No, but the information I provide may be used to inform the overall analysis**

**Participant’s signature**: __________________________**Date** _______________________

(Please print name)

I certify that I have followed all the study specific procedures described in the SOP for obtaining informed consent.

**Designee/investigator’s signature:** ____________________________ **Date** ____________

**Designee/investigator’s name:** _______________________________**Time** ____________

(Please print name)
