# Supplementary material for: Informing new or improved vector control tools for reducing the malaria burden in Tanzania: a qualitative exploration of perceptions of mosquitoes and methods for their control among the residents of Dar es Salaam
Source: Malar J. 2017 Oct 11;16:410. doi: 10.1186/s12936-017-2056-9 (PMC5637339; doi:10.1186/s12936-017-2056-9)
Supplement: Supplementary file 5 — Additional file 5. Informed consent for FGDs participants (1), English version (2) Swahili version. [file 12936_2017_2056_MOESM5_ESM.zip › Informed consent for FGDS- Swahili version.doc]

**Jina la Mradi:** Mbinu Mpya za Kudhibiti Wadudu Waenezao Magonjwa Afrika (African Vector Control : New Tools AvecNet )

**Lengo:** Kuboresha mbinu zilizopo au kupata mbinu mpya za kudhibiti mbu ili kupunguza tatizo la malaria katika bara la Afrika

**Taarifa ya mradi kwa washiriki wa majadiliano katika vikundi**

| **Taasisi Husika** | **Mhusika** |
| --- | --- |
| Taasisi ya Afya Ifakara | Christina Makungu |

**Utafiti huu unahusu nini?**

Madhumuni ya utafiti ni kuboresha au kupata mbinu mpya za kudhibiti mbu ili kupunguza maambukizi ya malaria. Kwa ujumla tunataka kujua wakazi wa eneo hili wanatumia njia gani kujikinga na mbu na sababu zinazopelekea kutumia njia hizo. Vilevile tutapenda kujua watu wanafikiri nini juu ya mbu, matatizo ambayo mbu wanasababisha au faida zinazoletwa na mbu. Uzoefu, mtazamo na maoni ya wakazi wa eneo hili yatasaidia kupata taarifa zitakazo tuwezesha kupata mbinu mpya au kuboresha mbinu zilizopo ili kupunguza malaria kwa kudhibiti mbu.

**Nini kitatokea?** Kama mtakubali kushiriki, tutakuwa na majadiliano ya pamoja katika kikundi. Mahojiano yatachukua saa moja mpaka saa moja na nusu. Tutapenda kuzungumza na ninyi juu ya mtazamo wenu dhidi ya mbu kama wanaleta madhara au faida yoyote katika eneo lenu. Tungependa pia kujua mtazamo wenu juu ya nani anawajibika kudhibiti mbu katika eneo lenu, njia mnazotumia kudhibiti mbu na sababu zinazopelekea matumizi ya njia hizo.

Mkikubali, maongezi hayo yatarekodiwa katika kanda ama kisahani (disc) lakini rekodi hizi zitaharibiwa mwishoni mwa mradi. Kama hamtapenda majadiliano kurekodiwa au kusitisha rekodi, mtafiti atafurahi kutekeleza ombi lenu. Mwishoni mwa utafiti, kila mshiriki atapewa taarifa rahisi na fupi ya mradi na matokeo ya utafiti.

**Madhara :** Hakuna madhara yoyote katika kushiriki utafiti huu.

**Faida kuu:** Hakuna faida binafsi atayopata mtu kwa kushiriki katika utafiti huu, lakini matokeo ya utafiti huu yatatusaidia kupata taarifa zitakazotuwezesha kudhibiti maambukizi ya malaria kwa kuboresha mbinu zilizopo au kupata mbinu mpya zinazokubalika katika jamii.

**Usiri:** Maelezo mtakayotupatia yatahifadhiwa kwa usiri kwa ajili ya mradi tu. Maelezo hayo yatahifadhiwa katika chumba salama kinachofungwa. Pia yatahifadhiwa katika kompyuta inayolindwa kwa neno la siri. Utaweza kuona maelezo uliyotupatia wakati wowote ukitoa taarifa yenye kueleza sababu za msingi za kutaka kufanya hivyo. Majina yenu hayatatumika kutambulisha maelezo yoyote uliyotupatia katika ripoti ama maandishi yoyote ya mradi. Kwa ridhaa maalumu, picha zitazopigwa wakati wa majadiliano zitatumika katika machapisho ya utafiti huu.

**Malipo:** Hakuna gharama kwa ushiriki wako katika utafiti huu. Hamtapatiwa malipo yoyote kwa kushiriki katika utafiti

**Kujitoa ushiriki:** Utakuwa huru kujitoa katika utafiti wakati wowote bila kulazimika kutoa maelezo ya kuamua kufanya hivyo na bila kupigwa faini yoyote kwa kufanya hivyo. Unaweza kujitoa kwa kuongea na mhusika wa mradi ama kwa maandishi.

**Nini kinachofuata**: Ushiriki wako katika utafiti huu ni wa hiari. Ukikubali kushiriki utaombwa kusaini fomu ya ridhaa. Na iwapo utataka kusitisha ushiriki wako katika utafiti huu unaombwa kutoa tarifa ya maneno au maandishi kwa watafiti na hakuna athari yoyote itakayokupata.

**Maswali:** Kama una maswali yoyote tafadhali kuwa huru kumuuliza mhusika yeyote wa utafiti.

**Anuani ya mtafiti/mhusika:** Christina Makungu

S.L.P 78373

Mikocheni, Kiko avenue

**Dar-es-salaam**

**Namba ya simu ya mkononi: 0688-423982**

**Fomu ya ridhaa kwa washiriki wa majadiliano ya vikundi**

Nathibitisha kwamba tumeelezwa vya kutosha madhumuni ya utafiti huu wenye jina la AvecNet. Tumeelewa madhumuni ya utafiti na tumepatiwa muda wa kutosha kuuliza maswali pia tumeridhishwa na majibu yaliyotolewa. Tunathibitisha kwamba tunaelewa kuwa takwimu zitakazokusanywa wakati wa majadiliano haya zitatumika kwa matumizi ya utafiti, lakini pia kwenye machapisho na ripoti. Tumeelewa pia kuwa ushiriki wetu katika utafiti huu ni wa hiyari, kwa hiyo tuna uhuru wa kujitoa katika utafiti huu. Tunatambua kuwa tunaweza kukataa kujibu maswali au kutoa taarifa, pia tunaweza kuomba majadiliano yasitishwe au kujitoa ushiriki bila kuathirika kwa namna yoyote.

**Sehemu hii itajazwa na mwezeshaji wa majadiliano**

**Tunakubali kushiriki katika majadiliano haya *tafadhali mwezeshaji atie tiki kwenye boksi mojawapo*** ** Ndiyo  Hapana**

**Tunakubali vinasa sauti vinaweza kuwa vinatumika kurekodi majadiliano kama sehemu ya utafiti *tafadhali mwezeshaji atie tiki kwenye boksi mojawapo***

** Ndiyo  Hapana**

**Tunakubali matumizi ya majina yasiyo halisi katika machapisho ya mradi huu *tafadhali mwezeshaji atie tiki kwenye boksi***

** Ndiyo , endapo taarifa yoyote itayotolewa itatutambulisha kwa majina yasiyo halisi**

** Hapana, lakini taarifa yoyote tutakayoitoa itatumika katika uchambuzi wa utafiti**

**Sehemu hii itajazwa na washiriki wawili kama mashahidi wa kundi**

Ninathibitisha kuwa ridhaa ya maneno imetolewa na kundi

**(Tafadhali weka sahihi au dole gumba ) ______________________________**

**Tarehe ____________ (ANDIKA JINA KWA HERUFI KUBWA)________________________________________**Ninathibitisha kuwa ridhaa ya maneno imetolewa na kundi **(Tafadhali weka sahihi au dole gumba) ________________________________________ Tarehe __________ (ANDIKA JINA KWA HERUFI KUBWA) _______________________________________Ninakiri kuwa nimefuata kwa umakini taratibu zote za zinazohusu namna ya kupata ridhaa ya washiriki kama zilivyoainishwa kwenye taratibu za mradi huu.**

**Sahihi ya mtafiti : ________________________________ Tarehe __________________Jina la Mtafiti : _________________________________**
